# Spectroscopic Study of the Molecular Structure of the New Hybrid with a Potential Two-Way Antibacterial Effect

**DOI:** 10.3390/molecules26051442

**Published:** 2021-03-07

**Authors:** Dorota Kowalczuk, Agata Gładysz, Monika Pitucha, Daniel M. Kamiński, Agnieszka Barańska, Bartłomiej Drop

**Affiliations:** 1 Department of Medicinal Chemistry, Faculty of Pharmacy, Medical University, Jaczewskiego 4, 20-090 Lublin, Poland; agata.gladysz@umlub.pl; 2 Independent Radiopharmacy Unit, Faculty of Pharmacy, Medical University, Chodzki 4A, 20-093 Lublin, Poland; monika.pitucha@umlub.pl; 3 Department of Chemistry, University of Life Sciences in Lublin, Akademicka 15, 20-950 Lublin, Poland; daniel.kaminski@poczta.umcs.lublin.pl; 4 Department of Informatics and Medical Statistics, Medical University, Jaczewskiego 4, 20-090 Lublin, Poland; agnieszkabaranska@umlub.pl (A.B.); bartlomiej.drop@umlub.pl (B.D.)

**Keywords:** ciprofloxacin–biguanide bismuth complex, metallo-organic compound, structure characterization, spectroscopic study, FTIR, NMR

## Abstract

Bacterial strains become resistant to almost all classes of antibiotics, which makes it necessary to look for new substitutes. The non-absorbable ciprofloxacin–biguanide bismuth complex, used locally, may be a good alternative to a conventional therapy. The purpose of this study was to study the structure of the proposed ciprofloxacin (CIP) -bismuth(III)—chlorhexidine (CHX) composite (CIP-Bi-CHX). The spectroscopic techniques such as UV-VIS (ultraviolet-visible) spectroscopy, FTIR (Fourier-transform infrared) spectroscopy and NMR (Nuclear Magnetic Resonance) spectroscopy were used for structure characterization of the hybrid compound. The performed analysis confirmed the presence of the two active components—CIP and CHX and revealed the possible coordination sites of the ligands with bismuth ion in the metallo-organic structure. Spectroscopic study showed that the complexation between Bi(III) and CIP occurs through the carboxylate and ketone groups of the quinolone ring, while CHX combines with the central ion via the biguanide moieties.

## 1. Introduction

Bacterial resistance to antimicrobial drugs might become one of the biggest threats to human health in the 21st century. The rapid global spread of Gram-positive and Gram-negative bacterial pathogens, that are resistant to currently available antimicrobial therapies marks the onset of a major global health crisis. Clinicians are forced to improve medical practices and procedures to combat the spread of antibiotic resistance, which is not easy at a time when access to antibiotics and chemotherapeutic agents is common [1,2,3]. The problem of the spread of bacterial resistance is also associated with the production and the antibiotic production wastewater, as well as with the wide application, excretion and penetration into the aquatic environment [4,5].

One way to solve this problem is to develop new antibiotics or new drug classes that delay the evolution of drug resistance. In recent years, the number of studies looking for new compounds with antibacterial properties has increased significantly. To this end, new molecules are synthesized in the hope of their potential antibacterial activity, compounds and substances naturally occurring in the environment are tested, or compounds with proven antimicrobial activity are combined to form hybrids, composites, or complexes [6]. Scientists make attempts to create drug complexes with metal ions [7,8,9], combine drugs with different range of activity [10], incorporate substances naturally occurring in the environment into the polymer matrix [11,12]. It is possible that combining two therapeutic agents may enhance their antibacterial properties or even give a new mechanism of antibacterial action to the resulting hybrid. Interactions between different components are governed by a variety of chemical bonds. The study of their properties can be a valuable tool to understand the influence of interactions between components on their properties [13]. The interesting approach is the development of new dual acting antibacterial combinations or compounds by the simultaneous administration of two different antimicrobial agents, or by combining two molecules of different antibiotics together through a chemical reaction to obtain a single molecule named a hybrid [10,14]. The advantage of this approach is the development of compounds that possesses often better biological activity or even more, that may be active to microorganisms that are resistant to both antibiotics.

Structure information of newly synthesized composites is important not only for the synthesis verification and determination of the structure–activity relationship (SAR), but also the entire development and registration process [15,16].

Nuclear magnetic resonance (NMR) spectroscopy and Fourier transform infrared spectroscopy (FTIR) are the commonly used methods for the confirmation of synthesis and structural characterization of a novel chemical framework [17,18,19,20,21,22,23,24,25,26,27,28,29,30,31,32,33]. The spectroscopic studies were used: (i) For the characterization of metal-complexes [17,18,19,20,21,22,24,32,33] such as metalloantibiotics [21,22], including metallofluoroquinolones [18,19,22,26,33] and metal complexes with chlorhexidine [20,24], (ii) for the analysis of the decomposition of metal-complexes [20], (iii) for the characterization of inclusion compounds [25], composites/mineral composites [27,28,31,32] and (iv) for the examination of interactions between the components [23]. Infrared (IR) spectroscopic method, especially the Attenuated Total Reflectance (ATR) technique and NMR spectroscopic measurements were often applied to confirm the coordination of the ligands to metal ions and to find out the possible coordination sites in the metallo-organic structures [17]. The NMR spectroscopic measurements and FTIR studies were also proposed for the confirmation of synthesis hybrids [30].

This article is intended to demonstrate the application of spectroscopic techniques (particularly ATR-FTIR and NMR) for the structure characterization of the new metal-drug composite that is targeted at the local treatment of wounds.

## 2. Results and Discussion

Based on the knowledge that the ligands in metal complexes can be the ions or neutral molecules containing the atoms with free electron pairs (in particular N, O, halogens) such as carboxylate [19], triethylenetetramine, bipyridine [18,19], biguanide [20,29], the new composites (hybrids) were synthetized by covalently combining fluoroquinolone antibiotics and the biguanide derivatives with metal ion.

We present the spectroscopic structure characterization of the selected hybrid containing ciprofloxacin (CIP, fluoroquinolone antibiotic, DNA gyrase inhibitor) and chlorhexidine (CHX, biguanide derivative, antiseptic, agent disrupting the cell membrane function) as ligands. The prepared dual acting hybrid with antibacterial and potentially anti-inflammatory properties are dedicated for the local treatment of wounds. Our previous research has shown that the ciprofloxacin -bismuth complex exhibits high anti-bacterial activity and a broad spectrum of activity [33].

Taking into account of the synthesis conditions, CIP and CHX ligands were bonded to bismuth atom in a molar ratio of 1: 1: 1. Thus, the following compositions of prepared hybrid were considered:

(a) Bi(CHX)2NH_3_: C(35.45%) H(4.46%) Bi(28.03%) Cl(9.51%) N(22.55%),
(b) Bi(CIP)(2NH_3_)2Cl: C(31.69%) H(3.60%) Bi(32.44%) Cl(11.01%) F(2.95%) N(10.87%) O(7.45%),
(c) Bi(CIP)(CHX): C(44.92%) H(4.35%) Bi(20.04%) Cl(6.80%) F(1.82%) N(17.46%) O(4.60%),
(d) Bi(CIP)(CHX)2CI: C(41.99%) H(4.25%) Bi(18.73%) Cl(12.71%) F(1.70%) N(16.32%) O(4.30%).

The results of elemental analysis (Table 1) showed that the structure of the composite [Bi(CIP)(CHX)2CI] is the most probable—the T-Student test showed no significant differences between the values found and the values calculated (*P* (0.12–0.95) > 0.05). Results also revealed that the reaction of CIP and CHX with bismuth salt in molar ratio of 1:1:1 gives the mixed ligand complex with stoichiometric 1:1:1. The obtained bismuth complexes are very slightly soluble in common organic solvents. Thus, the studies of electrolytic behavior of metal complex solutions, providing the insights into their nature and composition, were not successful. The melting point of the CIP-Bi-CHX composite was found to be 294.7 °C, while the melting points of the ligands, CIP and CHX, and their physical mixture, CIP + CHX, were found to be 255 °C, 134 °C and 225.7 °C, respectively.

**Figure 1:**
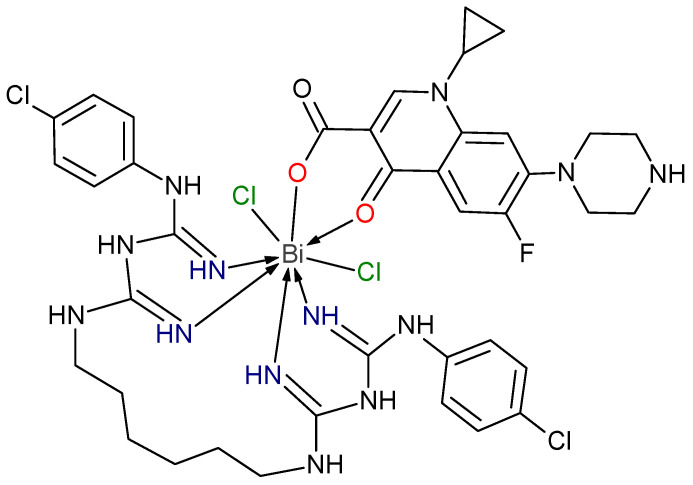
The probable structure of metal complex (generated by ACD ChemSketch Freeware).

Figure 1 shows the structure of the CIP-Bi-CHX composite, which was suggested from the above analysis. Similar structures have been proposed for antibiotic-based complexes [18,19,21], including quinolone complexes with the participation of bipyridine [18,19].

### 2.1. UV-VIS Analysis

**Figure 2:**
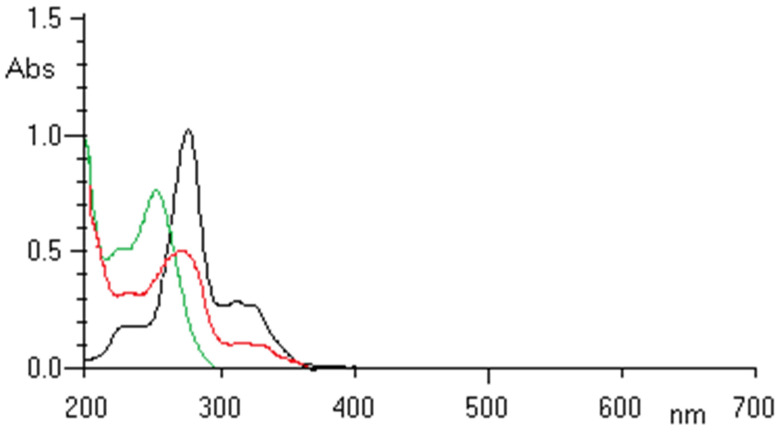
UV-VIS absorption spectra of ciprofloxacin (back line), chlorhexidine (green line) and ciprofloxacin-Bi-chlorhexidine composite (red line).

Chlorhexidine acetate shows absorption bands in the ultraviolet (UV) range at 208 nm and 259 nm. The spectrum of ciprofloxacin has a strong absorption band at 277.0 nm and two much weaker bands near 315.0 nm and 328.0 nm (Figure 2). The spectrum of the complex in the UV region shows the same characteristic bands as the spectra of the ligands, but the complex formation influences changes in the shape of the bands and their shifts. In the visible (VIS) region, the composite shows no absorption bands. Upon complexation with Bi(III), the bands of CIP in the region from 310 nm to 340 nm were modified and the major absorption band of CIP at 277.0 nm was shifted slightly to 272 nm and widened by overlapping with the main band of CHX. The observed changes suggest the formation of a six-member ring (complexation between Bi(III) and CIP via the carboxylate and ketone group) which disturb the conjugation system of the ciprofloxacin structure [23,33].

### 2.2. FTIR Analysis

**Figure 3:**
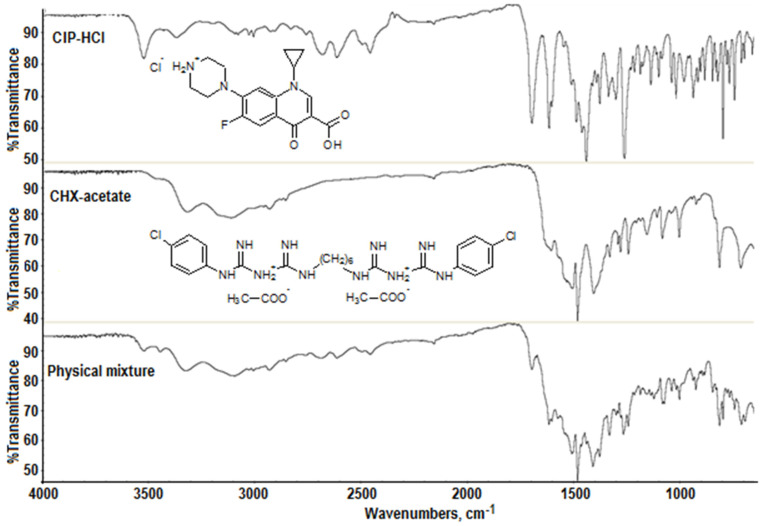
FT-IR spectra registered for CIP-HCl, CHX-acetate and their physical mixture (ν_max_/cm^−1^).

**Figure 4:**
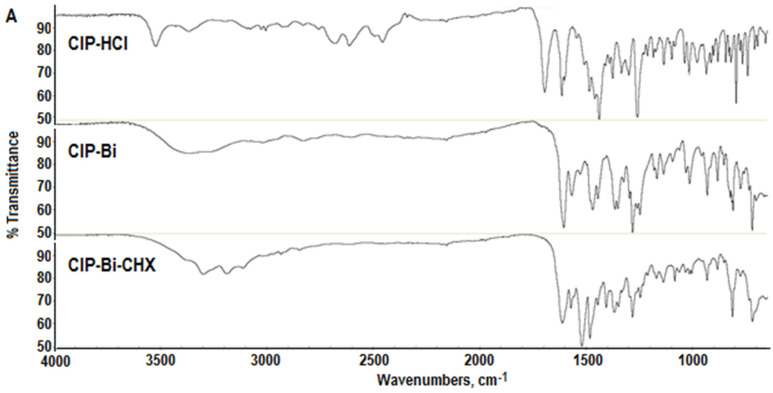

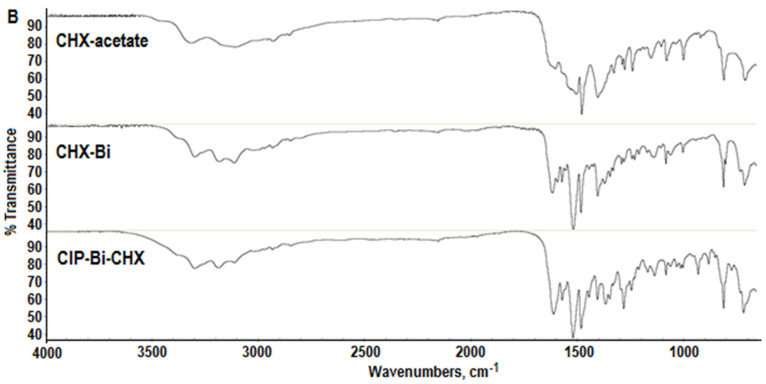
FTIR spectra obtained in the subsequent stages of the hybrid synthesis for method 1 (**A**) and method 2 (**B**).

A FTIR analysis was conducted for active substances (ligands) used for the synthesis of the hybrid—ciprofloxacin hydrochloride (CIP-HCI), chlorhexidine acetate (CHX-acetate), their physical mixture (CIP-HCI + CHX-acetate) (Figure 3) as well as the CIP-Bi-CHX composite, taking into account two methods of its synthesis and two synthesis stages: (i) The formation of simple complex (CIP-Bi or CHX-Bi), (ii) the formation of mixed ligand complex (CIP-Bi-CHX) (Figure 4). The recorded spectra were subjected to a comparative analysis which demonstrated the presence of vibrational bands characteristic for the functional groups occurring in the analyzed molecules (Table 2 and Table 3). The absorption bands obtained for CHX-acetate and CIP-HCl (Table 2 and Table 3) are in good agreement with previous literature [20,24,25,27,28] and [22,26,34], respectively.

The spectrum of the physical mixture of CIP and CHX salts is characterized by higher intensity when compared to individual substances with minor band shifts of functional groups, which can result from the intermolecular interactions of the said substances in the mixture (Figure 3, Table 2).

The IR spectrum of the ciprofloxacin-bismuth(III) complex shows changes suggesting the formation of a complex salt within the carboxyl and ketone groups in relation to the ligand, i.e., ciprofloxacin hydrochloride. The carboxyl group (COOH) in the CIP HCl molecule undergoes deprotonation, due to the substitution of hydrogen by the bismuth(III) ion, which results in the formation of an ionised carboxylate group (COO^−^) in the CIP-Bi molecule. As a consequence, the stretching vibration band of the hydroxyl group (v OH) disappears at 3528 cm^−1^, whereas the stretching vibration band of the carbonyl group (v C=O) in the carboxyl group undergoes a significant shift towards lower frequencies to 1702 cm^−1^. An intensive stretching vibration band appears at 1575 cm^−1^, characteristic of the carbonyl group in the carboxylate group (a change of the COOH group in the CIP molecule into COO- in the CIP-Bi molecule). A shift towards lower frequencies also occurs in the carbonyl spectrum of the ketone group—from 1623 cm^−1^ in the CIP molecule to 1614^−1^ in the CIP-Bi molecule—due to the formation of a coordinate bond between C=O and the bismuth ion. In addition, the IR spectrum of the CIP-Bi complex shows changes allowing the conclusion that the ciprofloxacin in the complex is a base rather than a hydrochloride. The asymmetric and symmetric stretching vibration bands of the ionised secondary amino group (ν as/s >NH_2_^+^) in the range of 2688–2465 cm^−1^ present in the CIP-HCl molecule disappear in the spectrum of the complex and the stretching vibration bands of the non-ionised secondary amino group (ν as/s >NH) appear at about 3400–3200 cm^−1^, which are significantly extended probably due to the formation of hydrogen bonds (the associated amino group) and/or the effect of molecular configuration in crystal.

The changes observed in the IR spectrum of the complex of chlorhexidine with the bismuth(III) ion, with regard to the CHX-acetate ligand, indicate the coordination of CHX to the Bi(III) ion by the nitrogen atoms of amino groups (-C=NH). In the spectrum of the CHX-Bi complex, the stretching vibration bands at 3195 cm^−1^ and 1625 cm^−1^ corresponding to the =NH and C=N groups, respectively, and the bending vibration band at 1520 cm^−1^ corresponding to the secondary amino groups (=NH), show a positive shift and significant intensification. Although it is also possible for the nitrogen to coordinate amino groups, numerous studies of the complex compounds of biguanides emphasize the precedence of the formation of complexes with metals by nitrogen from imino groups, probably due to an increase in stability via coupling π [29].

In the CIP-Bi-CHX hybrid, which can be considered as two metal-complexes, CIP-Bi and CHX-Bi, ciprofloxacin is present as a deprotonated anion. The COOH group in the CIP molecule undergoes deprotonation and forms a salt with bismuth(III) (bismuth carboxylate), stabilized with a coordinate bond originating from the oxygen of the ketone group adjacent to the said carboxyl group. The assumed concept of the bond between Bi(III) and the CIP molecule in the composite is confirmed by the changes observable during the analysis of the spectrum (Figure 4A):

- The disappearance of the stretching vibration band of the OH group at 3528 cm^−1^;
- The disappearance of the stretching vibration band of C=O in the COOH group at 1702 cm^−1^, and the appearance of the stretching vibration band of C=O in the COO^−^ group at 1580 cm^−1^;
- The shift and significant intensification of the stretching vibration band of the ketone group >C=O at 1621 cm^−1^ due to interference with the ν C=N band of chlorhexidine.

The metallic coordination center of the analyzed composite also contains chlorhexidine, which is coordinated to the Bi(III) ion through the nitrogen atoms of imino groups (-C=NH). The assumed hypothesis is confirmed by the following changes observable during the analysis of spectrum (Figure 4B):

- A positive shift (towards higher frequencies) from 3178 cm^−1^ in the CHX molecule to 3196 cm^−1^ in the composite molecule and an intensification of the stretching vibration band of the =NH group;
- A positive shift from 1611 cm^−1^ in the CHX molecule to 1621 cm^−1^ in the composite molecule, and a significant intensification of the stretching vibration band of the C=N group, probably indicating a loosening of the bond due to its coordination to the metal ion [29];
- A positive shift from 1515 cm^−1^ in the CHX molecule to 1520 cm^−1^ in the composite molecule, and a significant intensification of the stretching vibration band of the N=H group.

In addition, the IR spectrum of the composite shows changes leading to the conclusion that ciprofloxacin and chlorhexidine are present in the composite in a non-ionised form. The asymmetric and symmetric stretching vibration bands of the ionised secondary amino group (ν as/s >NH_2_^+^) in the range 2688–2465 cm^−1^, present in the CIP HCl molecule, disappear in the composite, as does the stretching vibration band of the acetate group (ν as/s COO^−^) present in the CHX acetate molecule at about 1536 cm^−1^ (Table 3).

### 2.3. ^1^H-NMR and ^13^C-NMR Analyses

**Figure 5:**
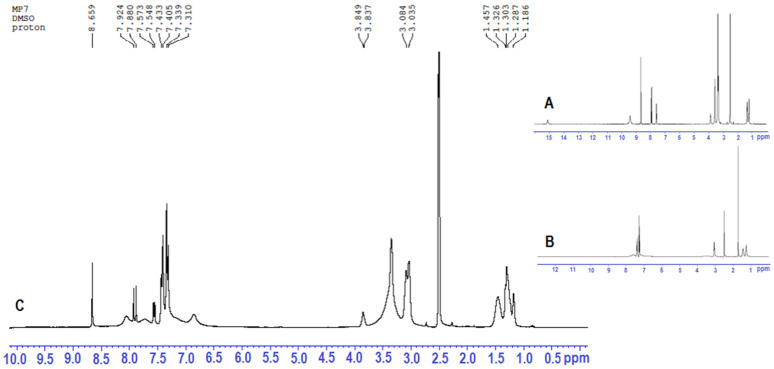
The ^1^H-NMR spectra of the parent forms of ligands: CIP-HCl (**A**), CHX-acetate (**B**) and the tested CIP-Bi-CHX composite (**C**).

**Figure 6:**
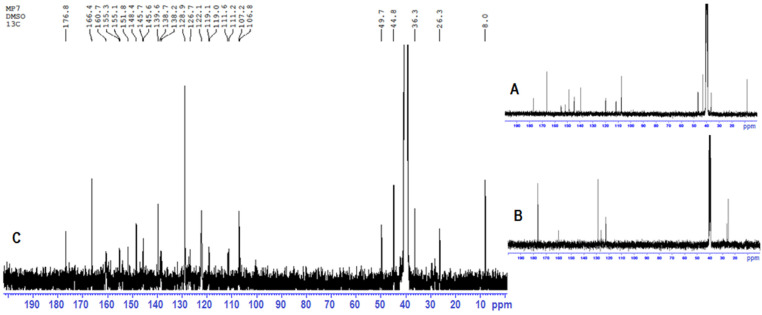
The ^13^C-NMR spectra of the parent forms of ligands: CIP-HCl (**A**) and CHX-acetate (**B**) and the tested CIP-Bi-CHX composite (**C**).

**Figure 7:**
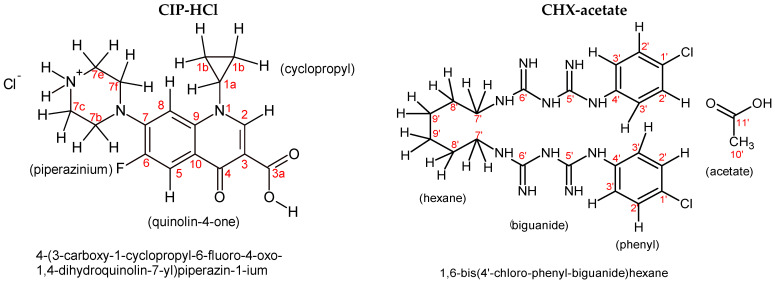
Structural formulas of CIP-HCl and CHX-acetate (generated by ACD ChemSketch Freeware).

The NMR spectra of the CIP-Bi-CHX composite (Figure 5 and Figure 6) indicate the presence of ciprofloxacin and chlorhexidine (Figure 7), as they contain signals characteristic of those substances (Table 4 and Table 5). The obtained ^1^H-NMR (Table 4) and ^13^C-NMR (Table 5) signals are in agreement with the spectral database for organic compounds [35] and available literature [36,37,38,39,40] for CHX-acetate and CIP-HCl, respectively. A comparative analysis of the NMR spectra also indicates the CIP and CHX bonding through the bismuth atom, owing to the appearance of significant changes in the composite spectrum, in relation to the same signals in the spectra of the parent substances. The changes involve:

- Differences in the multiplicity of the band;
- Differences in the band intensity (mainly signal reduction);
- Band broadening;
- Significant band shifts;
- Bands disappearance.

These changes indicate a strong interference into the molecules of the parent substances, with the possibility of forming a hybrid structure—forming covalent and coordinate bonds. 

The ^1^H-NMR spectrum of the composite show proton bands characteristic of ciprofloxacin at 1.19, 1.30–1.33, 3.84–3.85, and 7.55–8.66 ppm. When compared to the CIP, the CIP-Bi-CHX composite shows changes in the chemical shifts (Δ0.025–0.054 ppm) of the aromatic hydrogen atoms towards lower values in the quinolin-4-one ring, probably caused by the coordination of the Bi(III) ion by the oxygen atom of the 4-ketone group, and the formation of a covalent bond with the 3-carboxyl group, which is confirmed by the disappearance of the carboxyl group hydrogen signal at 15.14 ppm, due to the substitution of the carboxyl group proton with the bismuth atom. The similar changes in chemical shifts have been seen previously with the mixed ligand metal complexes [32]. The ^1^H-NMR spectrum of the composite shows the disappearance of the proton band of the >NH_2_^+^ group in the piperazinium ring, which is present in the CIP-HCl molecule [39]. This fact supports the assumption that CIP is present in the composite in its non-ionised form.

The ^1^H-NMR spectrum of the composite also shows the presence of proton bands characteristic of chlorhexidine at 1.29, 1.46, 3.04–3.08, 7.31–7.43 ppm, which underwent critical shifts after the incorporation of CHX into the composite structure. The changes particularly involve multiplicity and chemical shifts (Δ0.015–0.052 ppm) in the aromatic hydrogen atoms in the CHX phenyl rings towards higher shift values, and which are, with a high probability, the consequence of Bi(III) ion coordination to the nitrogen atoms of the biguanide groups directly connected with the aryl centers of the CHX molecule. In addition, the ^1^H-NMR spectrum of the composite shows the disappearance of the signal of the -CH_3_ group of the acetate molecule, which suggests that CHX is present in the composite in its non-ionised form.

**Scheme 1:**
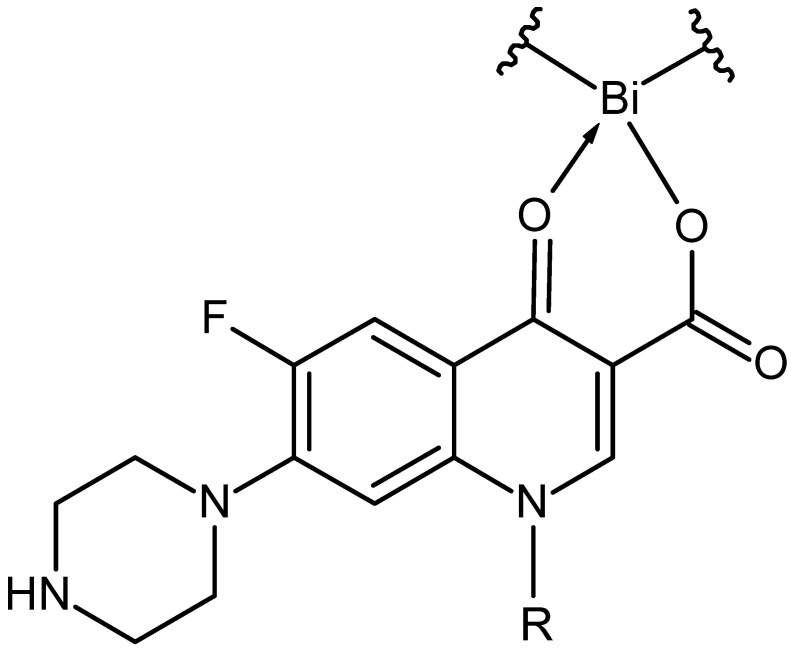
Six-membered ring formed probably by complexation of ciprofloxacin.

Changes in the ^13^C-NMR spectra of the composite, in relation to the spectra of the non-bonded substances, involve band shifts and band disappearances, and are observed in similar areas, as in the ^1^H-NMR spectra. When compared to CIP, the CIP-Bi-CHX composite indicates significant changes in the chemical shifts in the aromatic carbon atoms in the quinolin-4-one ring. The observed behaviour of the carbon signals might be caused by the inductive effect due to the participation of the 4-ketone group and 3-carboxyl group in the complexation of the Bi(III) ion (Scheme 1) with the formation of a six-membered ring [23]. The conclusion arrived at on the basis of the NMR analysis, stating that the coordination of metal ions with fluoroquinolones through the carboxyl and carbonyl group, occurring at positions 3 and 4 of the quinolin-4-one, respectively, is consistent with the available literature [23,41,42,43], as well as with the data obtained by interpreting the FTIR spectra.

Significant changes of the bands of hydrogen and carbon atoms in the piperazine ring of the composite might suggest that nitrogen atoms in this region are coordination locations [40,44], which is, however, unlikely, due to important evidence indicating the coordination of the Bi(III) ion with the COOH and C=O groups. In addition, the capability of forming a complex both by the COOH and C=O groups and the N-H group of piperidine [44] should also be rejected due to the ligand proportions in the composite (1:1:1, respectively for CIP, Bi and CHX). Therefore, the modifications of resonance corresponding to both the hydrogen and the carbon atoms in the piperazine ring after the incorporation of CIP into the composite might involve the deprotonation of the nitrogen atom, as well as intramolecular forces with other atoms of composite in the spatial configuration of its molecule and the interactions resulting from the configuration of the composite molecules in crystal, e.g., the interaction N-H…O(N), C-H…O(N), C-H (N-H)…π (aromatic system) [44,45,46].

**Scheme 2:**
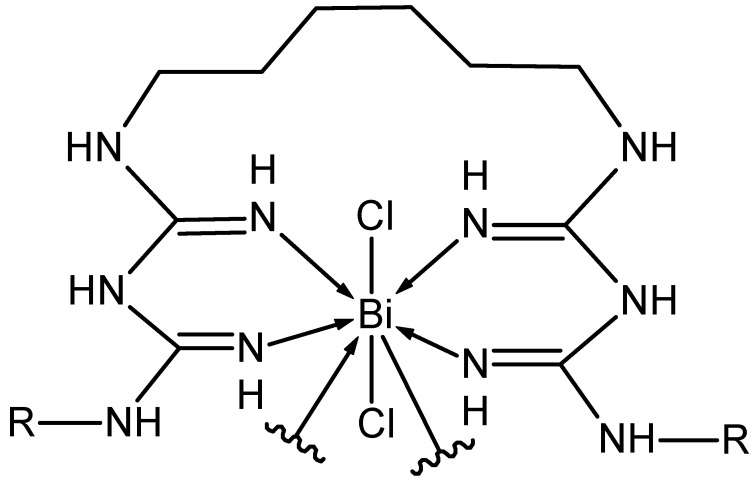
The suggested complexation of chlorhexidine.

In comparing the ^13^C-NMR spectrum of the composite and CHX, the greatest changes in chemical shifts can be observed for the carbon atoms of the aromatic area, and mainly in the biguanide group (bonded with the C4′ carbon of the aromatic area); the signal of the imine C6′ carbon (C=NH) atom moveds towards higher shift values, from 160.07 to 160.74 ppm, and the signals of C5′ and C4′ carbon atoms are not visible in the spectrum due to their low intensity. The said modifications might be the result of coordination of the Bi(III) ion with the nitrogen atoms of the imine groups (C=NH) of the biguanide groups (NH-C(=NH)-NH-C(=NH)-NH) of the CHX molecule (Scheme 2), as indicated by the available papers [47].

Furthermore, the ^13^C-NMR spectrum of the composite reveals the disappearance of the signals of the CH_3_ and COOH groups, which are present in the acetate group, indicating the deionisation of the CHX molecule in the composite.

### 2.4. X-ray Diffraction

**Figure 8:**
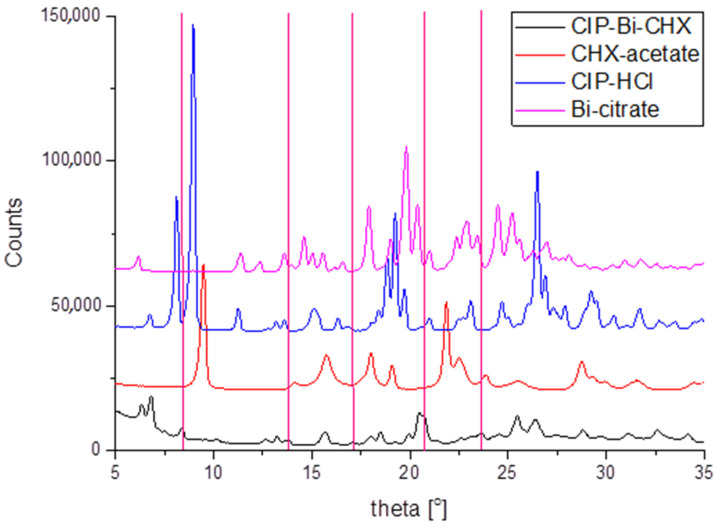
Diffraction patterns of CIP-Bi-CHX composite and the parent forms of ligands: Bi-citrate, CIP-HCl, CHX-acetate. For clarity presented patterns are shifted of 20,000 counts. The red vertical lines indicate a new phase of CIP-Bi-CHX.

The determination of X-ray crystal structures of the complexes was impossible because the efforts to obtain the suitable crystal were not successful. Thus, the X-ray powder diffraction measurements were performed for the phase identification of a crystalline material. Figure 8 shows powder diffraction data for the main product CIP-Bi-CHX and substrates used in synthesis. It can be seen that the main compound contains impurities of CHX-acetate, CIP-HCl, and Bi-citrate. The new phase of CIP-Bi-CHX can be characterized by weak reflections indicated by red vertical lines. The synthesized material has also amorphous content characterized by a broad background peak around 30°.

## 3. Materials and Methods

Ciprofloxacin (CIP) hydrochloride, chlorhexidine (CHX) diacetate, and bismuth(III) citrate (Bi) were obtained from Sigma-Aldrich Co. Ethanol, 35–38% hydrochloric acid, citric acid, and other reagents and solvents of analytical grade were acquired from Avantor Performance Materials Poland S.A. The water used in the experiments was double-distilled.

### 3.1. General Methods

^1^H and ^l3^C-NMR spectra were recorded on a Bruker Avance 300 spectrometer (Bruker BioSpin GmbH, Rheinstetten, Germany). The compounds (20 mg) were dissolved in DMSO-d6 (0.6 mL). In the case of sample slightly dissolved, the supernatant was decanted. Chemical shifts were reported in parts per million (ppm, Δscale) relative either to internal standard (TMS) or residual solvent peak. The number of scans used for registration was variable depending on weight of substance and quality of the signal (i.e., signal to noise). In the case of ^13^C-NMR it was 8000 scans.

FTIR (Fourier transform infrared spectroscopy) analysis was performed on a Thermo Scientific Nicolet 6700A spectrometer (Waltham, MA, USA) equipped with a deuterated triglycine sulfate detector (DTGS/KBr) and a versatile Attenuated Total Reflectance (ATR) sampling accessory with the diamond crystal plate. The FTIR spectra averaged over 32 scans were recorded in the spectral range of 600–4000 cm^−1^ at spectral resolution of 4 cm^−1^ using OMNIC 8.1 computer software (Thermo Fisher Scientific Inc., Waltham, MA, USA). The FTIR spectra were collected in the same way using the same pressure. The spectral intensities were compared in an absorbance mode by overlaying the spectra and measuring the absorbance values at a particular wavelength.

The absorption spectral measurements were carried out on a UV-Vis Hitachi U-2001 spectrophotometer (Hitachi Instrument Inc., Tokyo, Japan) controlled by UV Solutions software using 1 cm matched quartz cells. The examined solutions were prepared in dilute hydrochloric acid at the concentration of 10 μg/mL. The ultraviolet spectra were recorded in the range 200–700 nm.

The powder X-ray diffraction measurements were conducted on an Empyrean, PANalytical diffractometer using Cu anode which produced CuKα radiation (λ = 0.15406 nm). The measurements were performed in the 2θ angle range from 20° to 90°. The radiation source was equipped with a mirror to cut-off the CuKb radiation. The input slit was ½ mm and both detector and source were equipped with soller slits. The signals were recorded using a PixelX detector.

The elemental analyses were performed using a Perkin Elmer 2400 CHNS/O elemental analyzer. The chlorine was determined by the Schöniger procedure.

Melting points were recorded on a MP90 Melting Point System (Mettler-Toledo International Inc., Greifensee, Switzerland).

### 3.2. Preparation of the CIP-Bi-CHX Composite

The ciprofloxacin-bismuth-chlorhexidine (CIP-Bi-CHX) composite was synthesized according to the patent procedure no 235823 [48] by dissolving bismuth(III) salt in dilute hydrochloric acid, then mixing with chlorhexidine and ciprofloxacin in the ratio 1:1:1. The substances were added sequentially: first—CIP, later—CHX (method 1) or first—CHX, later—CIP (method 2). The syntheses were carried out with stirring at 40 °C for 15 min. Next the reaction medium was basified to precipitation with dilute ammonium hydroxide and allowed to stand at room temperature for 12 h. The pale yellowish precipitate was filtered, washed with water-ethanol (1:1 *v*/*v*) solution and dried in a desiccator under vacuum. Bi(CIP)(CHX)2Cl, molecular formula: C_39_H_47_BiCl_4_FN_13_O_3_, formula weight: 1115.67 g/moL, composition: C(41.99%) H(4.25%) Bi(18.73%) Cl(12.71%) F(1.70%) N(16.32%) O(4.30%).

## 4. Conclusions

The detailed spectroscopic characterization of the molecular structure of the new hybrid confirmed the presence of ciprofloxacin and chlorhexidine and revealed the possible coordination sites. Special attention was given to the interactions between the functional groups of the ligands and the bismuth ion in the relation to the parent forms of complexing agents. Based on the commonly used spectroscopic techniques and the literature data, the probable structure of the synthesized composite has been suggested. The applicability of the composite, the properties and healing advantages will be presented in subsequent papers.

It should also be emphasized that the conducted analyses have also shown the special role of spectroscopic techniques in the evaluation of the molecular structure of new compounds being potential drug candidates.

## 5. Patents

Results from the work reported in this manuscript are two patents [48,49].

## Figures and Tables

**Table 1:** Percentage of elements in the CIP-Bi-CHX hybrid.

Parameter	C	H	N	Cl
Found value, % (n = 6)	41.72	5.09	15.68	13.64
Minimum	37.16	4.56	15.12	12.67
Maximum	46.85	5.69	16.06	14.72
Variance	12.9246	0.1763	0.1285	0.5036
Standard deviation	3.5951	0.4199	0.3585	0.7096
Relative standard deviation, %	8.62	8.25	2.29	5.20
Calculated value, %	41.99	4.25	16.32	12.71
T-Student test, P = 95%	t = −0.07	1.85	−1.65	1.21
	P = 0.9466	0.1232	0.1601	0.2800

**Table 2:** The characteristic vibration bands registered for CIP-HCl, CHX-acetate and their physical mixture (ν_max_/cm^−1^).

Main Bands of Vibrations of Functional Groups	CIP-HCl	CHX-Acetate	Physical Mixture CIP-HCl + CHX-Acetate
CIP-HCl			
ν as/s (O-H in COOH)	3528m, 3375w		3525w
ν as/s (C-H in Ar)	3085w		about 3044w
ν as/s (C-H in CH_2_-cyclopropane)	3013w		3038w
ν as/s (C-H alk)	2937w		2939w
ν as/s (>NH_2_^+^)	2688m		2693m
ν (C=O; COOH)	1702s		1704 m
ν (C=O; ketone)	1623s		1623s
ν (C=C in Ar) + δ (NH)	1552–1445s		about 1495s
ν as/s (C-O)	1267s		1273 s
CHX-acetate			
ν (-NH) Alk–NH–Ar/(Alk)_2_NH		3324m	3329m
ν (=NH)		3178m	about 3180m
ν as/s (>NH_2_^+^)		3115m	3101m
ν (C=N)		1611s	1608s
ν as/s (COO- in acetate)		1536m	1538m
δ (NH)		1515s	1516s
ν (C=C) in Ar		1488s	1492s
ν (C–N)		1249m	1250m

Vibration type: ν—stretching vibration (as-asymmetric, s-symmetric vibration), δ—deformation vibration. Vibration intensity: vs—very strong, s—strong, m—moderate, w—weak; Ar—aryl, Alk—alkyl, aliph—aliphatic.

**Table 3:** The structural and spectral parameters of the parent forms of ligands: CIP-HCl, CHX-acetate, their complexes with bismuth(III): CIP-Bi, CHX-Bi and CIP-Bi-CHX hybrid (ν_max_/cm^−1^).

Main Bands of Vibrations of Functional Groups	CIP- HCl	CIP-Bi Complex	CHX-Acetate	CHX-Bi Complex	CIP-Bi-CHX Hybrid
CIP-HCl					
ν (O-H in COOH)	3528m, 3375w	No peak			No peak
ν as/s (>NH)	-	3362m			3383m
ν as/s (C-H in Ar)	3085w	about 3100w			No detected *
ν as/s (C-H in CH_2_)	3013w	3026w			No detected *
ν as/s (C-H in CH_3_)	2937w	2838w			2936w
ν as/s (>NH_2_^+^)	2688m	No peak			No peak
ν (C=O; COOH)	1702s	No peak			No peak
ν (C=O; ketone)	1623s	1614s			1621vs (int.)
ν as/s (COO-)	-	1575s			1580s
ν (C=C in Ar) + δ (NH)	1552–1445s	1533–1454s			1491s
ν as/s (C-O)	1267s	1291s			1291s
CHX-acetate					
ν (-NH) Alk–NH–Ar/(Alk)_2_NH			3324m	3307s	3307s
ν (=NH)			3178m	3195s	3196s
ν as/s (>NH_2_^+^)			3115m	3120s	3117m
ν (C=N)			1611m	1625vs	1621vs (int.)
ν as/s (COO^−^ in acetate)			1536m	No peak	No peak
δ (NH)			1515s	1520vs	1520vs
ν (C=C) in Ar			1488s	1492s	1491s
ν (C–N)			1249m	No detected *	No detected *

Vibration type: ν—stretching vibration (as-asymmetric, s-symmetric vibration), δ—deformation vibration. Vibration intensity: vs—very strong, s—strong, m—moderate, w—weak. Abbreviations: int.—intensification of intensity as a result of overlapping; Ar—aryl, Alk—alkyl, aliph—aliphatic; *—hard-to-detect peak.

**Table 4:** The values of chemical shifts for the hydrogen atoms of the parent forms of ligands: CIP-HCl and CHX-acetate, and the studied CIP-Bi-CHX composite. (+)—change in chemical shift towards higher ppm values; (−)—change in chemical shift towards lower ppm values; *—signal difficult to detect due to its low resolution.

H-Atom	Chemical Shifts for the Hydrogen Atoms (δ, ppm)			Change in Chemical Shift (∆δ, ppm)
	CIP-HCl	CHX-Acetate	CIP-Bi-CHX Composite	CIP-Bi-CHX Composite
**1b** (CIP, *cis* CH_2_ cyclopropyl)	1.194		1.186	Δ0.008 (−)
	1.203			
9′ (CHX, CH_2_ hexane)		1.263	1.287	Δ0.024 (+)
**1b** (CIP, *trans* CH_2_ cyclopropyl)	1.317		1.303	Δ0.014 (−)
	1.337		1.326	
8′ (CHX, CH_2_ hexane)		1.444	1.457	Δ0.013 (+)
10′ (CHX, CH_3_ acetate)		1.715		No peak
7′ (CHX, CH_2_ hexane)		3.041	3.035	Δ0.006 (−)
		3.064		
		3.086	3.084	
**7** (CIP, CH_2_ piperazinium)	3.568		No detected *	No detected *
**1a** (CIP, CH cyclopropyl)	3.854		3.837	Δ0.017 (−)
	3.866		3.849	
	3.878			
2′ (CHX, CH phenyl)		7.258	7.310	Δ0.052 (+)
		7.281	7.339	
		7.288		
3′ (CHX, CH phenyl)		7.390	7.405	Δ0.015 (+)
			7.433	
**8** (CIP, CH quinolin-4-one)	7.600		7.548	Δ0.052 (−)
	7.625		7.573	
**5** (CIP, CH quinolin-4-one)	7.934		7.880	Δ0.054 (−)
	7.977		7.924	
**2** (CIP, CH quinolin-4-one)	8.684		8.659	Δ0.025 (−)
**>NH_2_^+^** (CIP piperazinium)	9.432			No peak
**3a** (CIP, COOH)	15.141			No peak

**Table 5:** Values of chemical shifts for the carbon atoms of ligands: CIP-HCl and CHX-acetate and the studied CIP-Bi-CHX composite. (+)—change in chemical shift towards higher ppm values; (−)—change in chemical shift towards lower ppm values.

C—Atom	Chemical Shifts for the Carbon Atoms (δ, ppm)			Change in Chemical Shift (∆δ, ppm)
	CIP-HCl	CHX-Acetate	CIP-Bi-CHX Composite	CIP-Bi-CHX Composite
**1b** (CIP, CH_2_ cyclopropyl)	8.091		8.048	Δ0.043 (−)
10′ (CHX, CH_3_ acetate)		25.286		No peak
9′ (CHX, CH_2_ hexane)		26.365	26.391	Δ0.026 (+)
**1a** (CIP, CH cyclopropyl)	36.465		36.369	Δ0.096 (−)
**7c/7e** (CIP, CH_2_ piperazinium)	42.950		44.887	Δ1.937 (+)
**7b/7f** (CIP, CH_2_ piperazinium)	46.776		49.790	Δ2.952 (+)
	46.838			
**8** (CIP, CH quinolin-4-one)	107.288		106.814	Δ0.474 (−)
**3** (CIP, =C< quinolin-4-one)	107.411		107.234	Δ0.177 (−)
**5** (CIP, CH quinolin-4-one)	111.501		111.298	Δ0.203 (−)
	111.808		111.610	
**9** (CIP, =C< quinolin-4-one)	119.763		119.079	Δ0.684 (−)
	119.867		119.181	
3′ (CHX, CH phenyl)		122.467	122.159	Δ0.308 (−)
1′ (CHX, C-Cl phenyl)		126.180	126.732	Δ0.552 (+)
2′ (CHX, CH phenyl)		128.689	128.928	Δ0.239 (+)
**10** (CIP, =C< quinolin-4-one)	139.554		138.282	Δ0.799 (−)
			138.755	
			139.661	
**7** (CIP, =C< quinolin-4-one)	144.537		145.641	Δ1.104 (+)
	144.675		145.774	
**2** (CIP, CH quinolin-4-one)	148.697		148.483	Δ0.214 (−)
**6** (CIP, C-F quinolin-4-one)	151.697		151.815	Δ0.118 (+)
	155.005		155.127	Δ0.122 (+)
			155.375	
6′ (CHX, biguanide)		160.072	160.741	Δ0.669 (+)
**3a** (CIP, COOH)	166.334		166.454	Δ0.120 (+)
11′ (CHX, COOH acetate)		176.391		No peak
**4** (CIP, C=O)	176.839		176.811	Δ0.028 (−)
	176.872			

## Data Availability

The data presented in this study are available in this article.

## Sample Availability

Not available.
